# Supplementary figures and images for: Detection of neoplastic-immune hybrid cells with metastatic properties in uveal melanoma
Source: Biomark Res. 2024 Jul 20;12:67. doi: 10.1186/s40364-024-00609-6 (PMC11264923; doi:10.1186/s40364-024-00609-6)

# UMM059

A

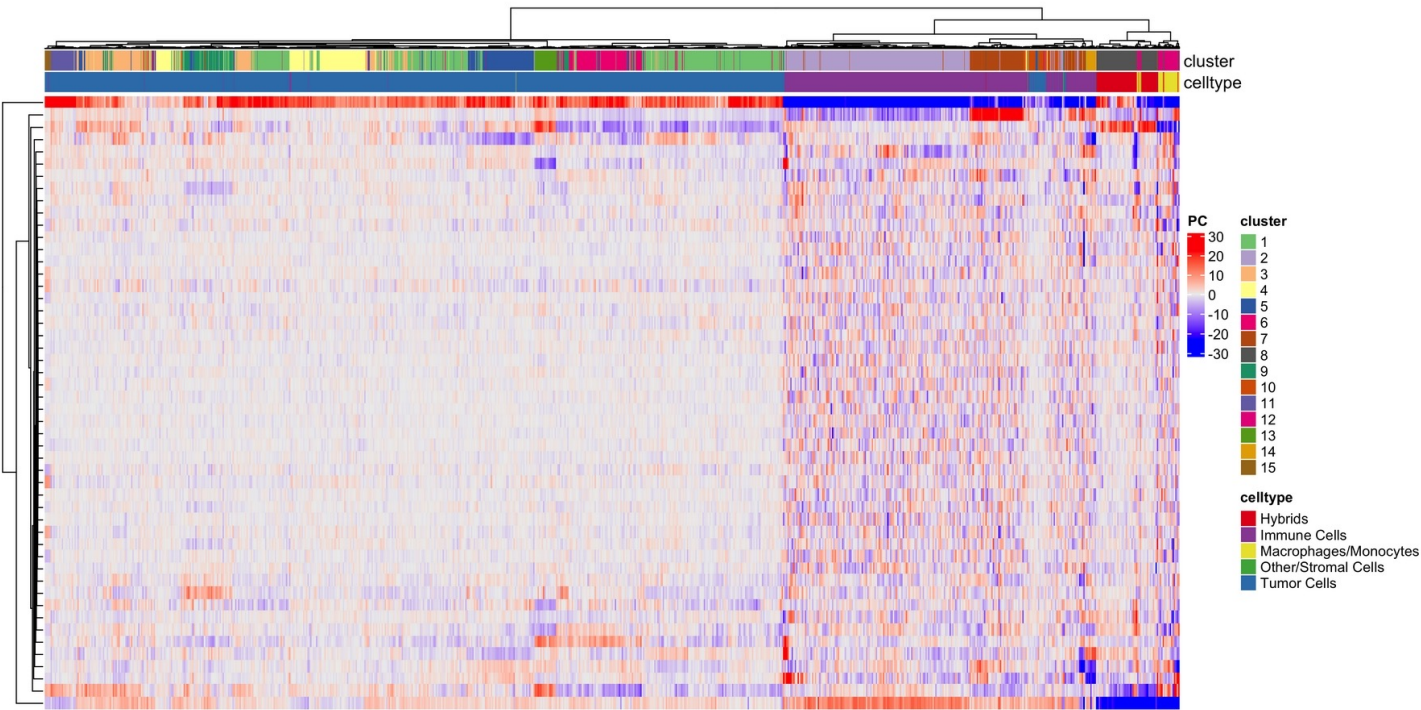

# UMM063

A

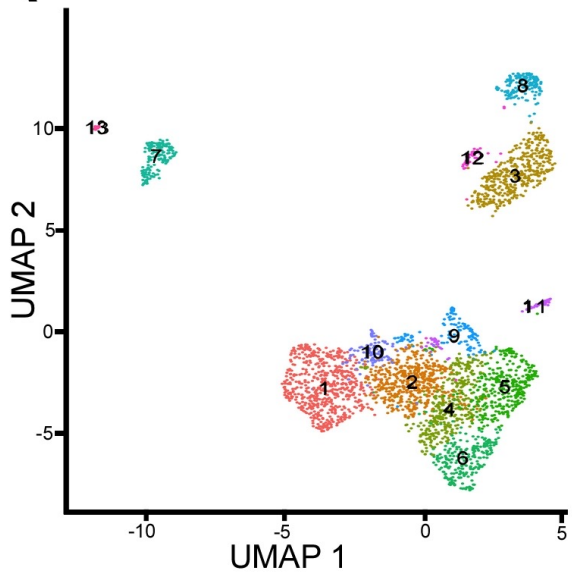

B

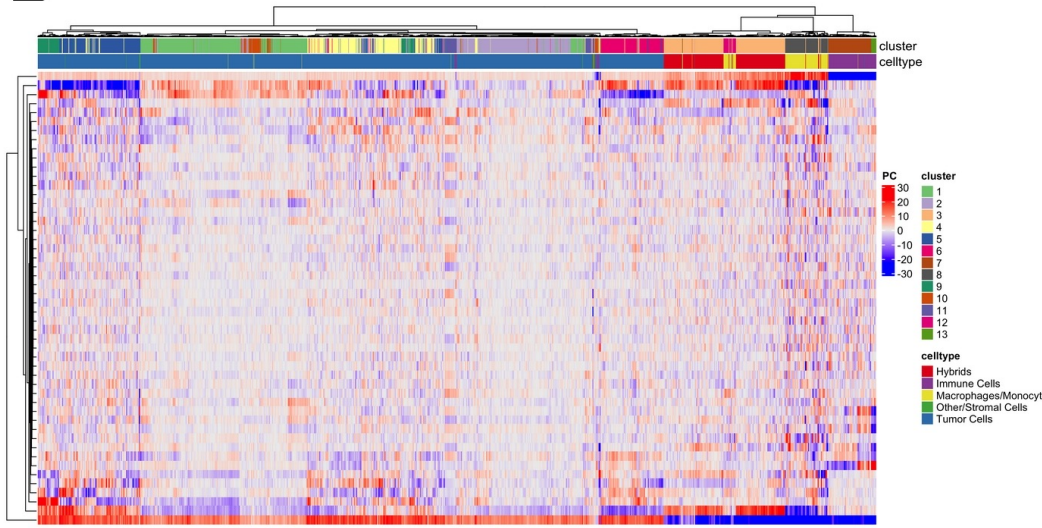

C

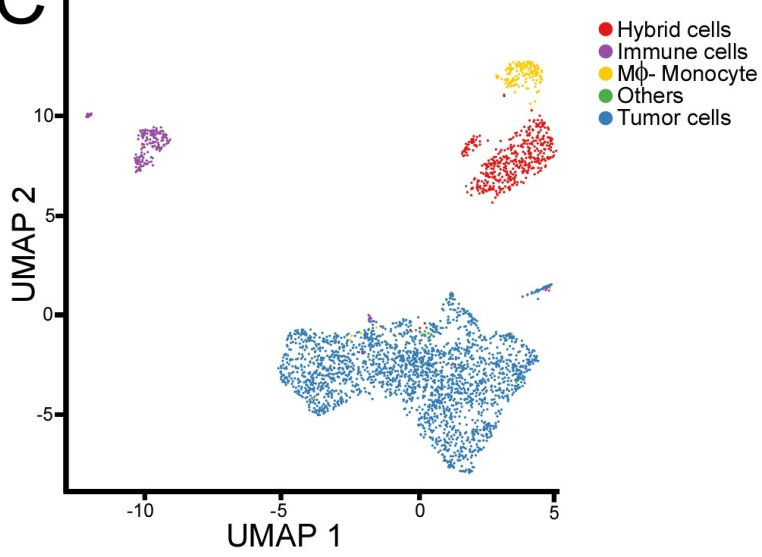

D

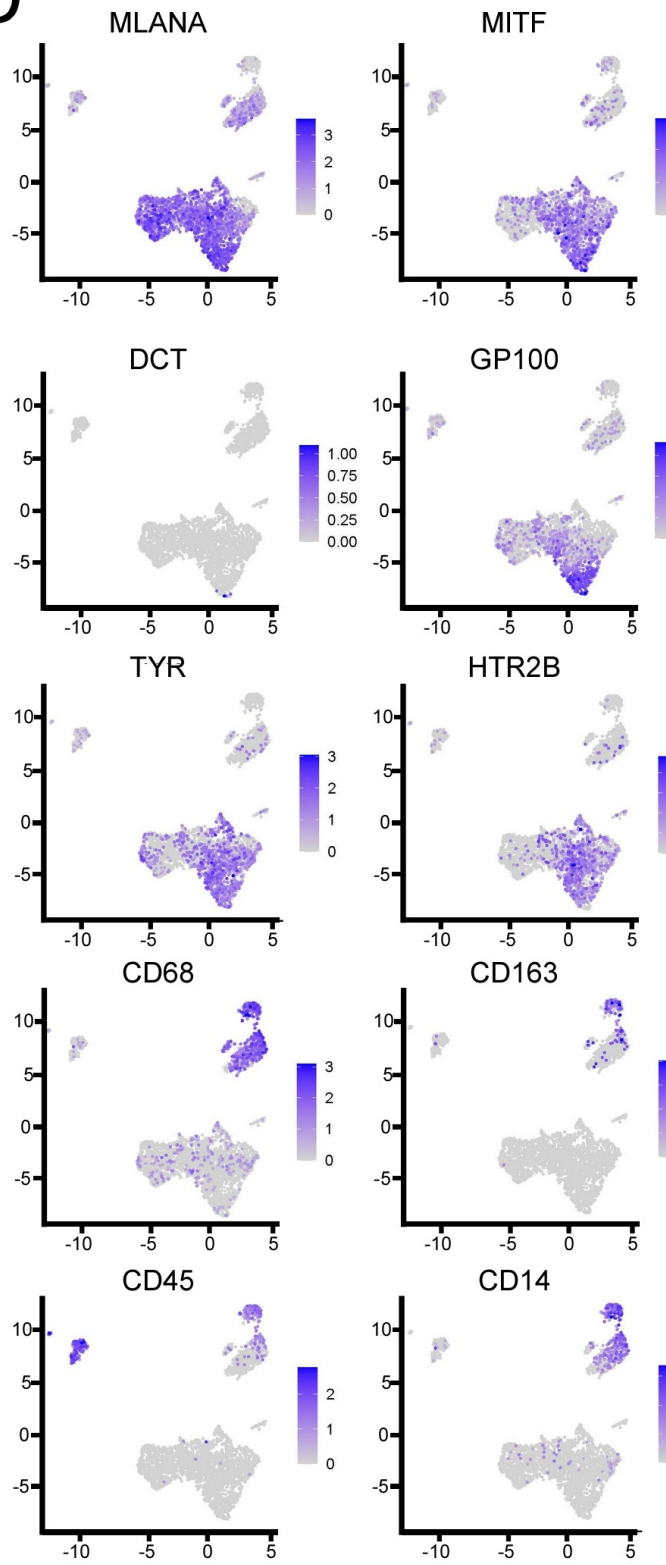

E

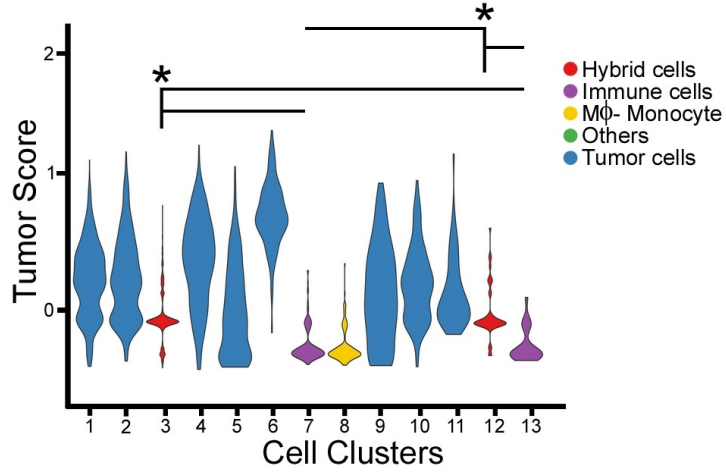

F

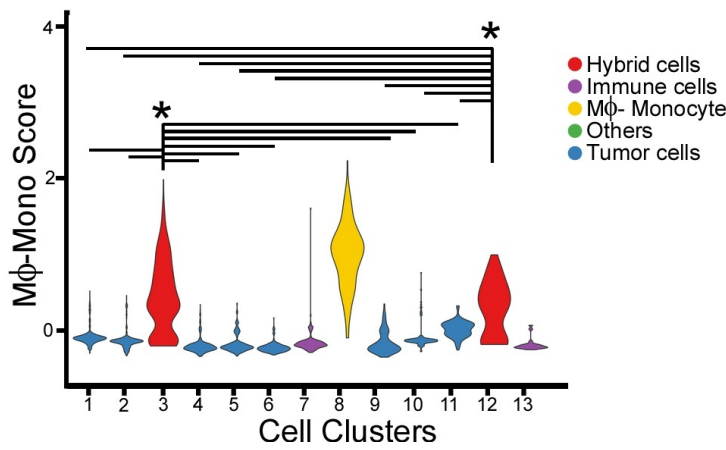

# UMM064

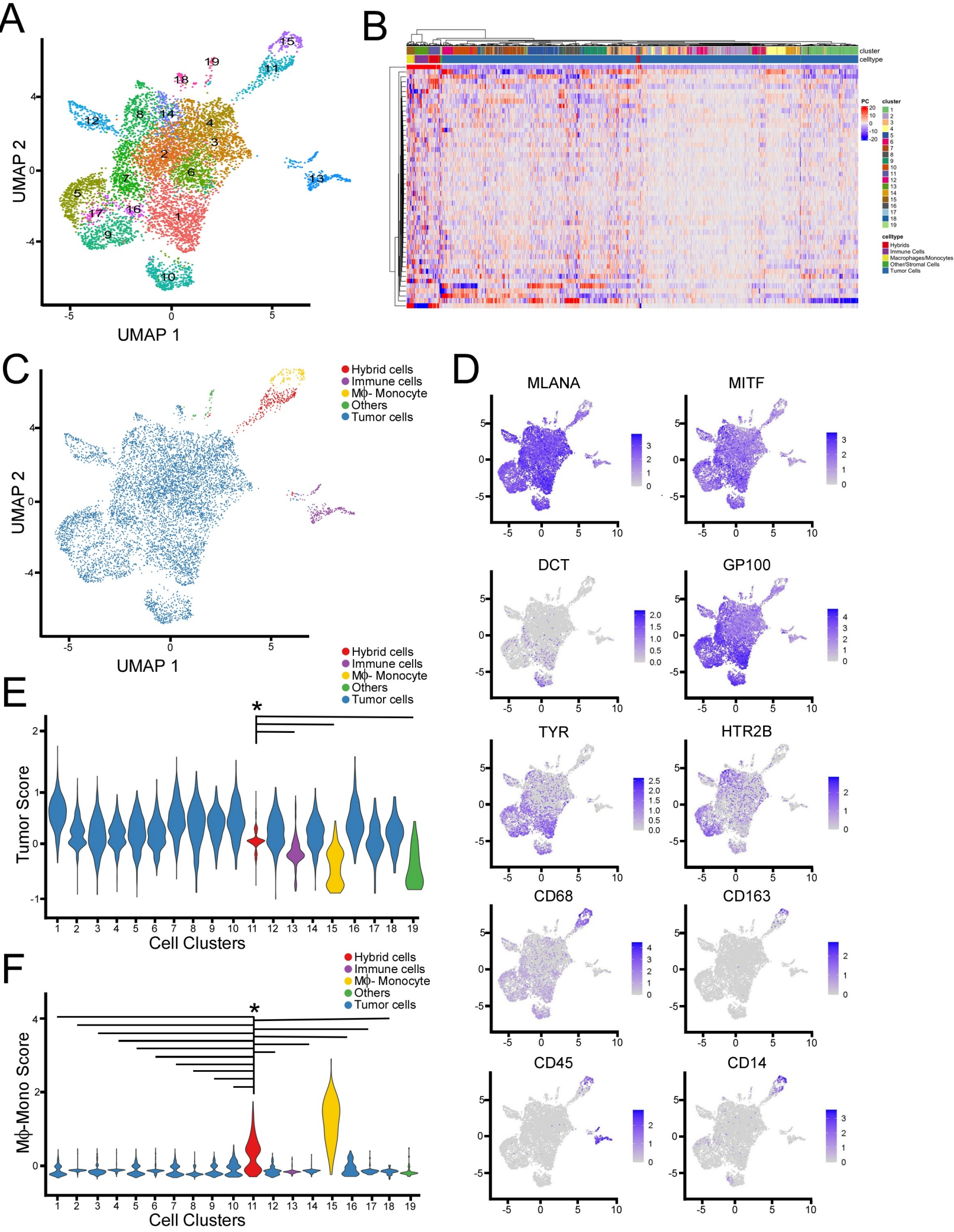

UMM065

A

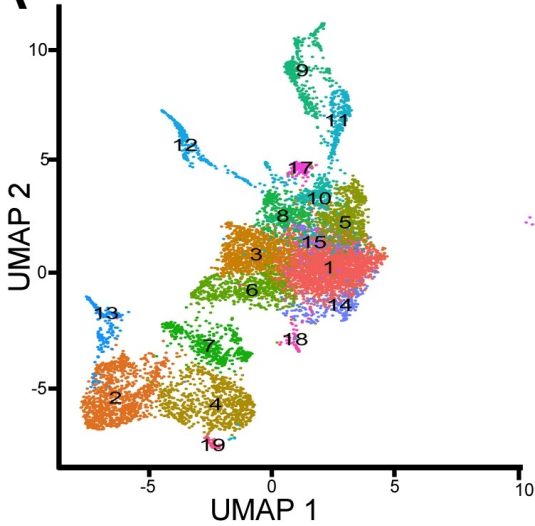

B

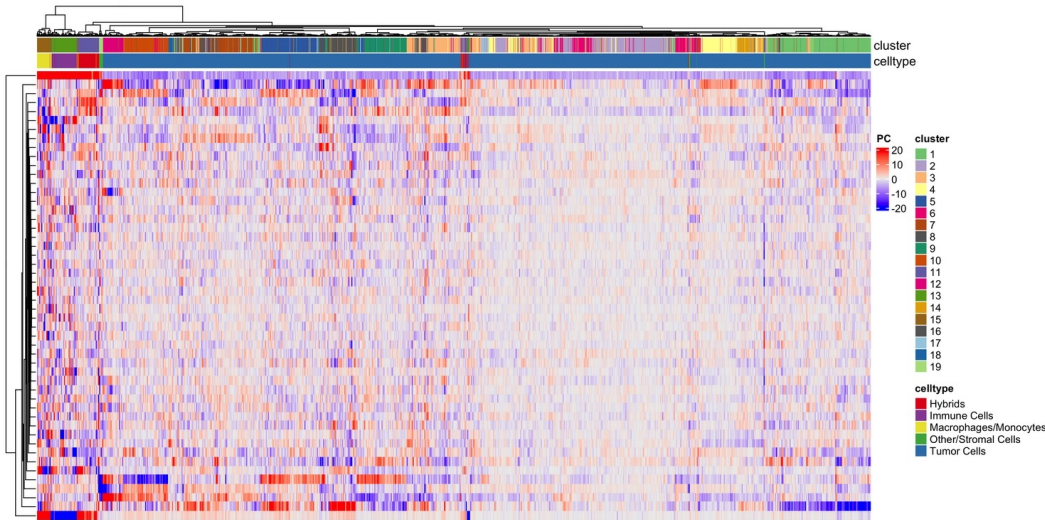

C

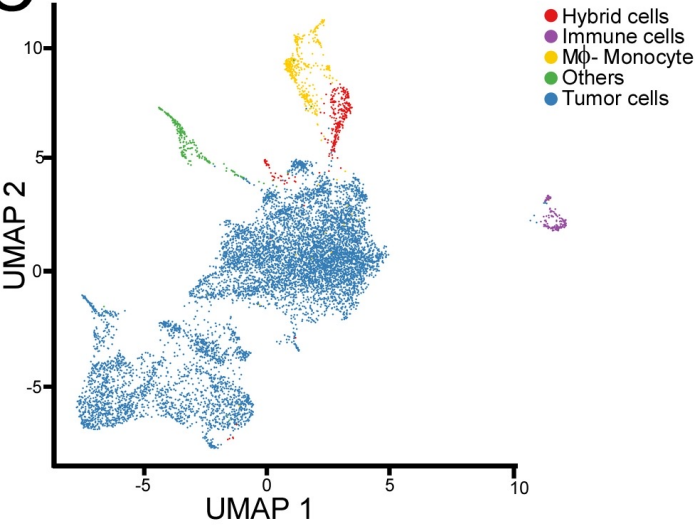

D

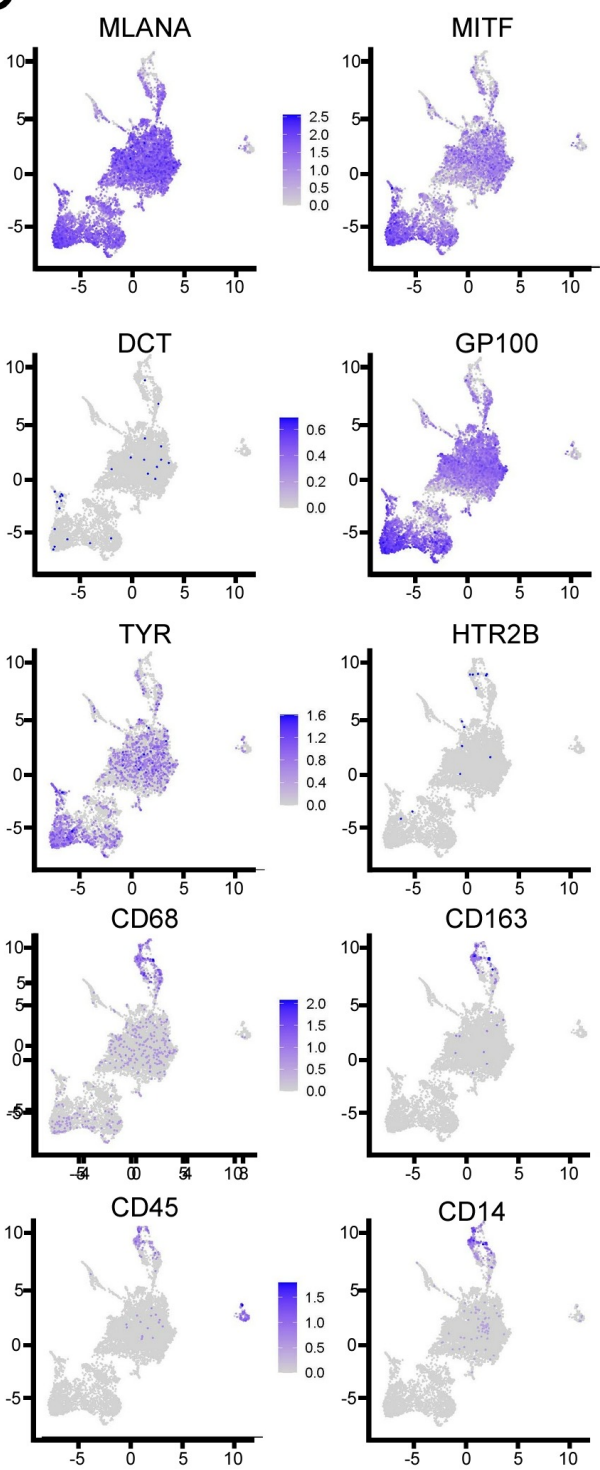

E

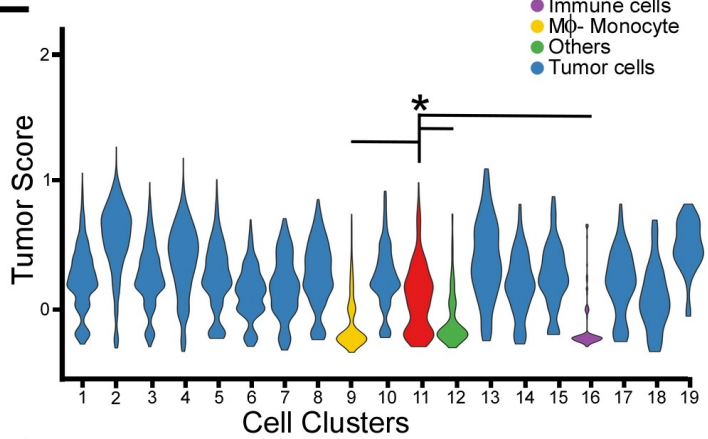

F

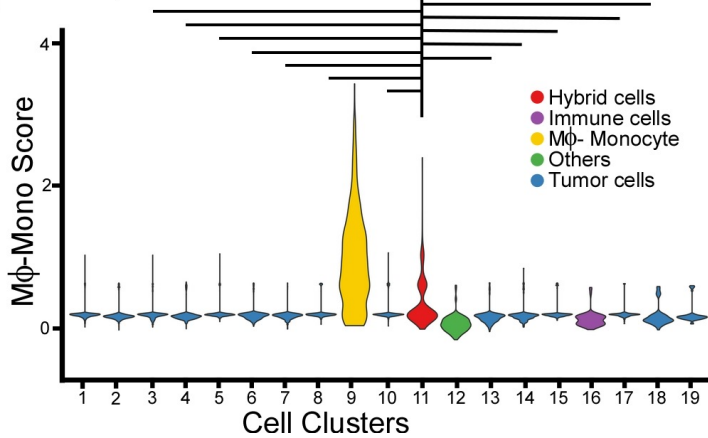

# UMM066

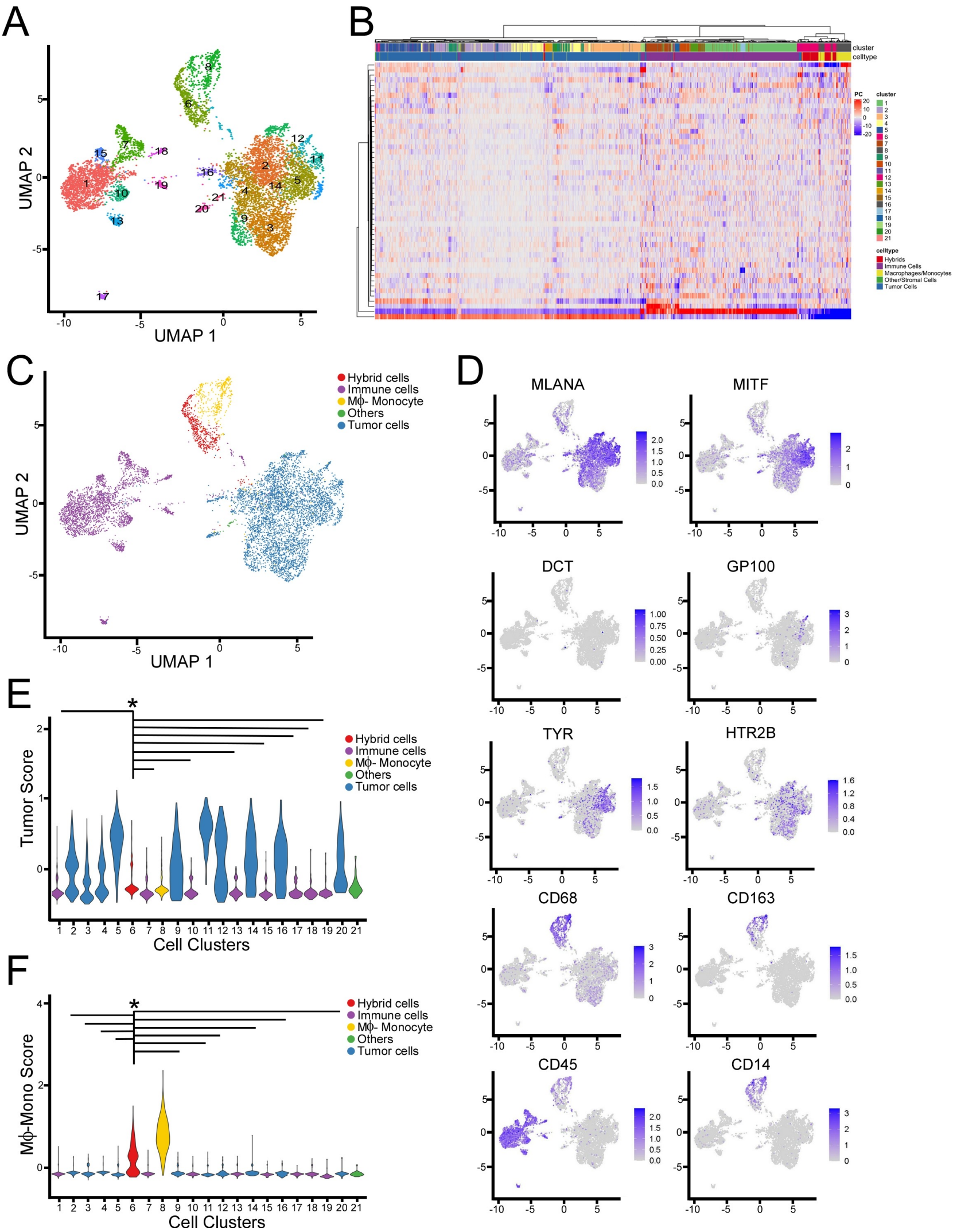

Supplement: Supplementary file 1 — Additional File 1:(UMM059) Hybrid cell identification from UM scRNA-seq dataset. A) Hierarchical clustering of scRNA-seq dataset for patient UMM059. [file 40364_2024_609_MOESM1_ESM.pdf]

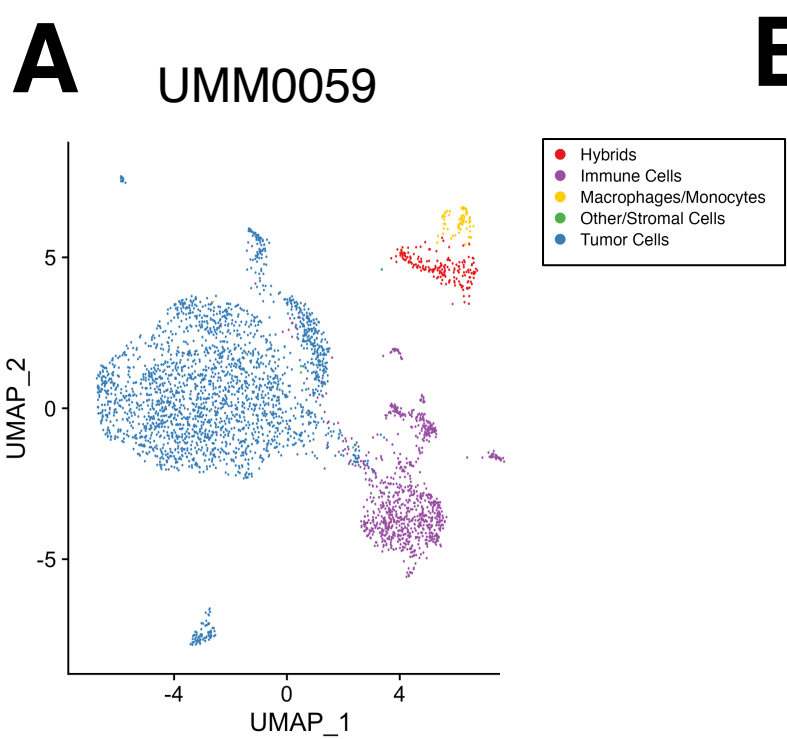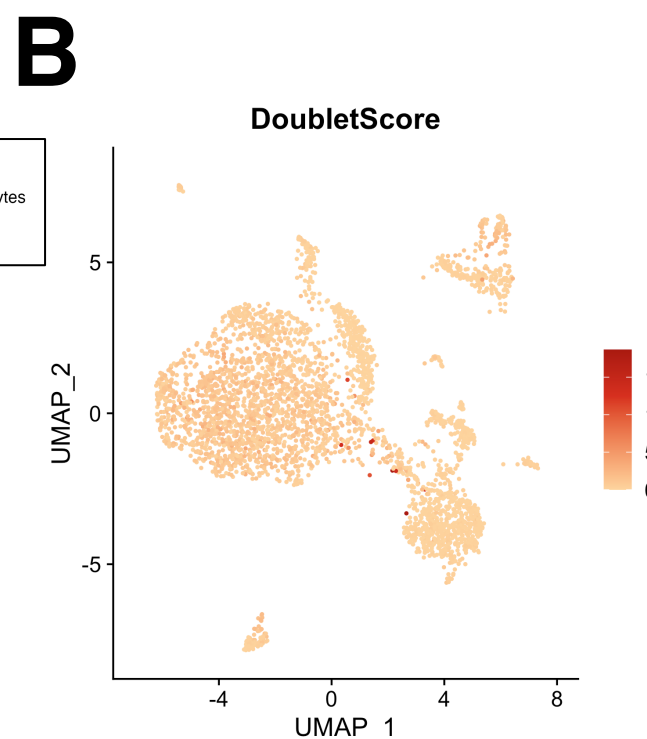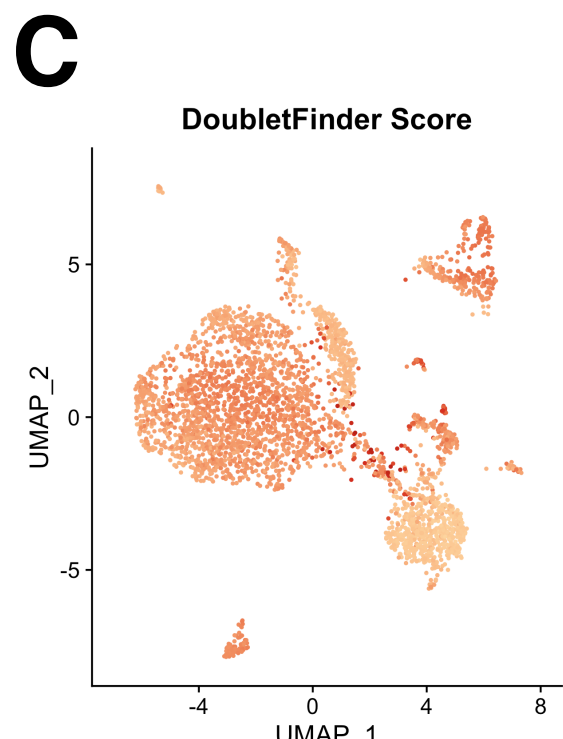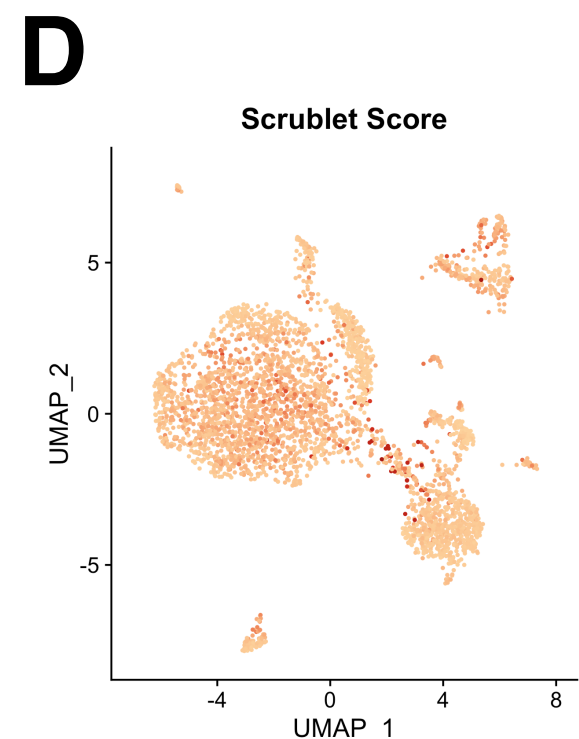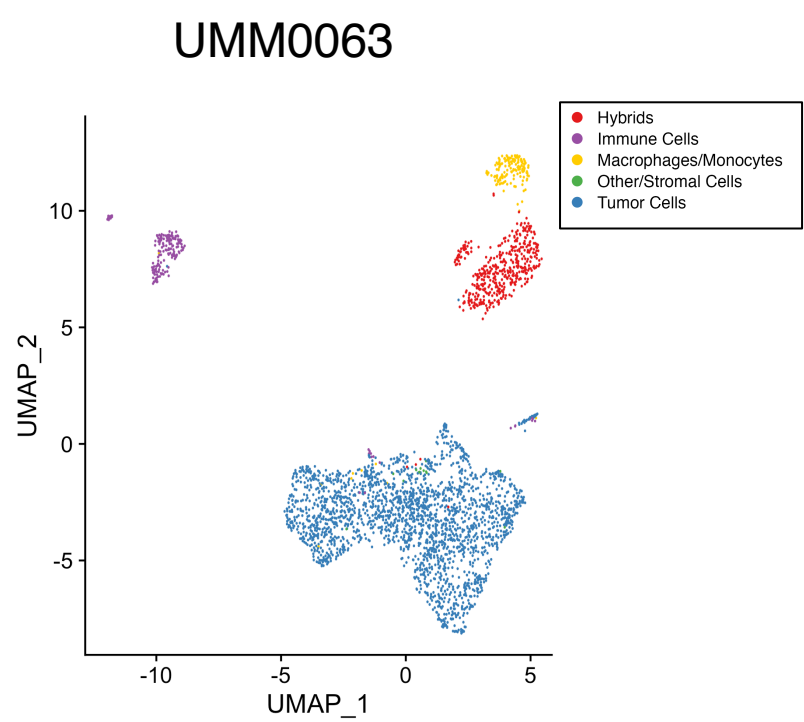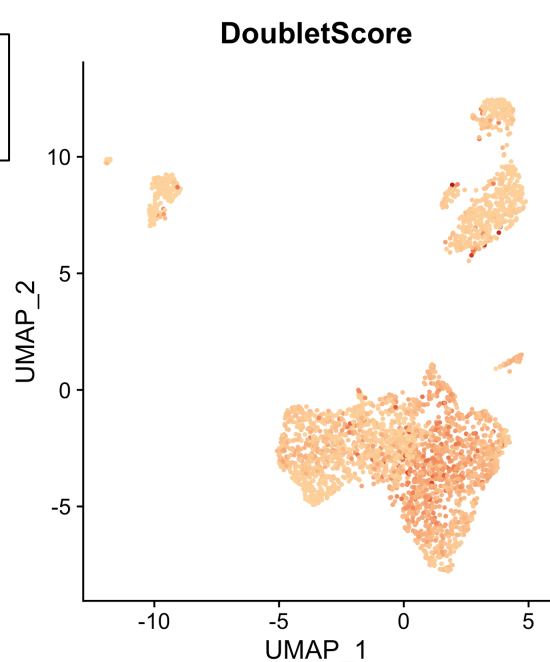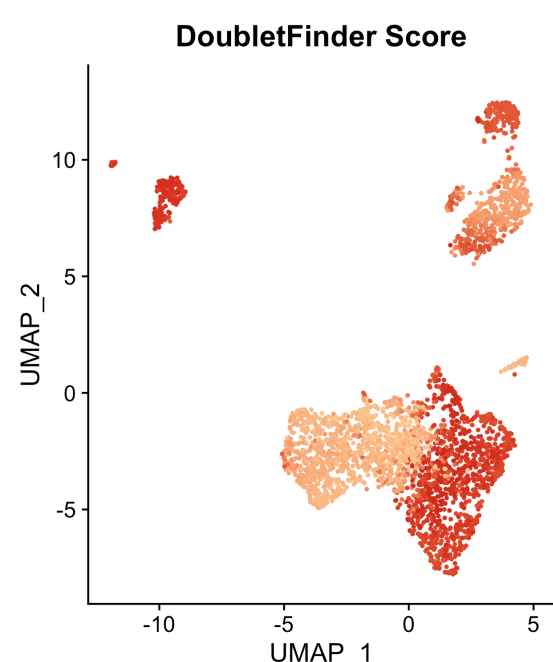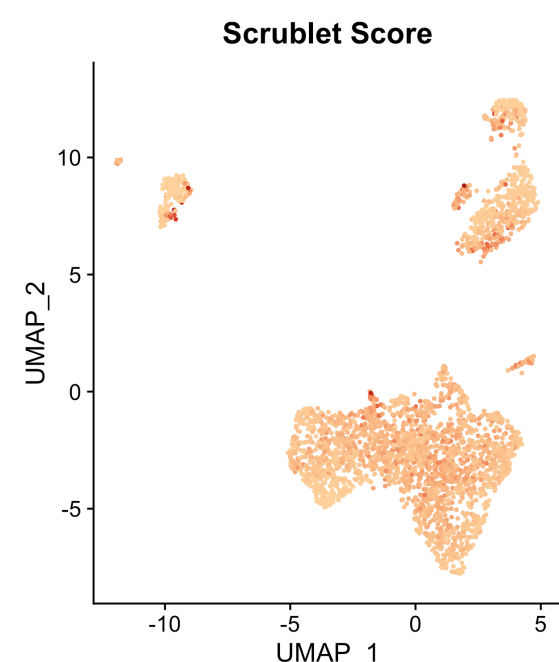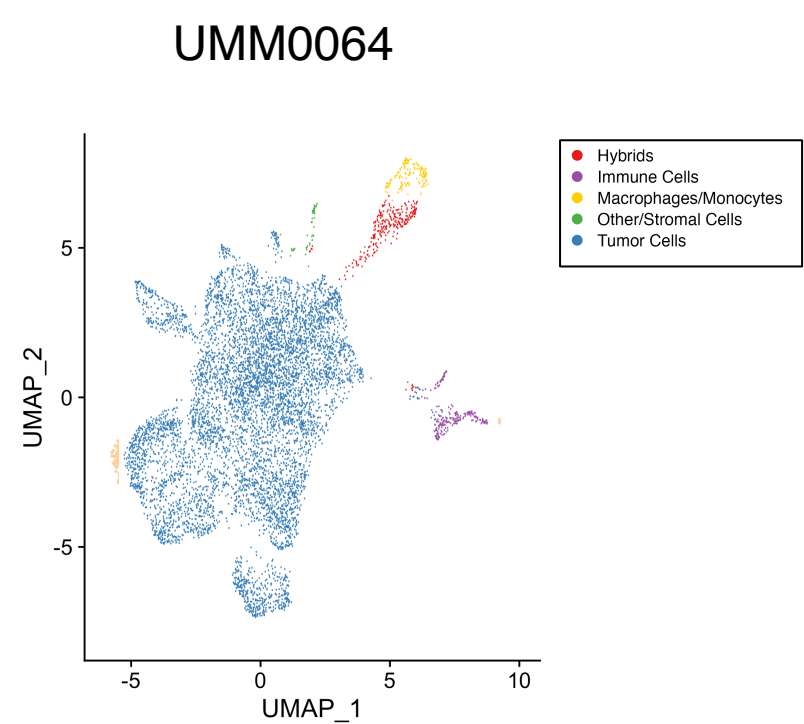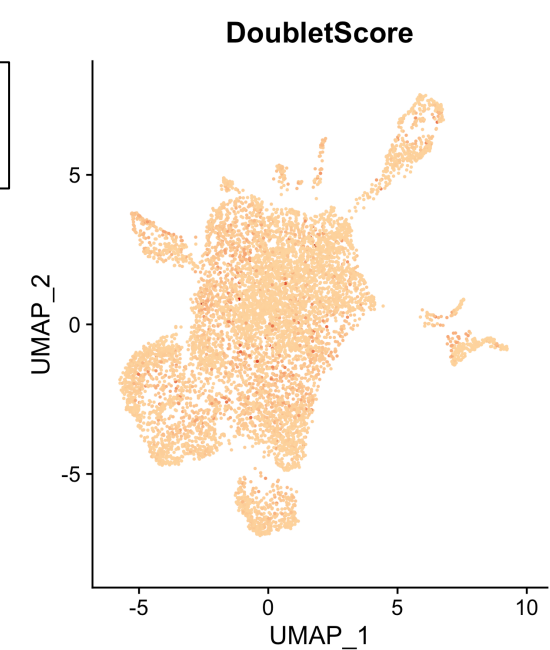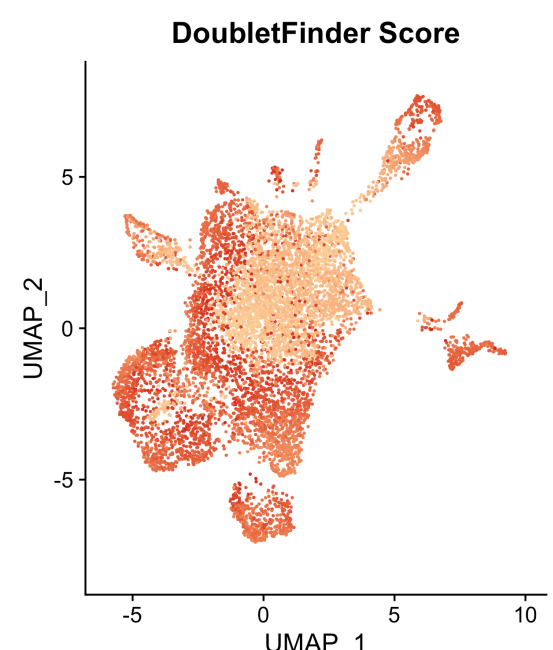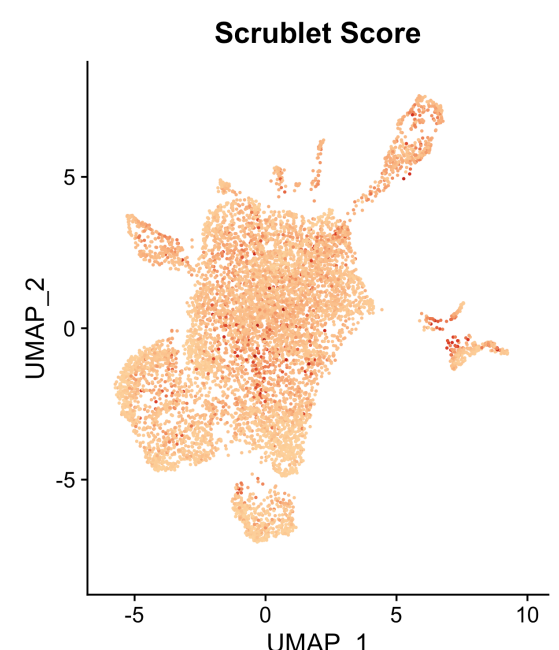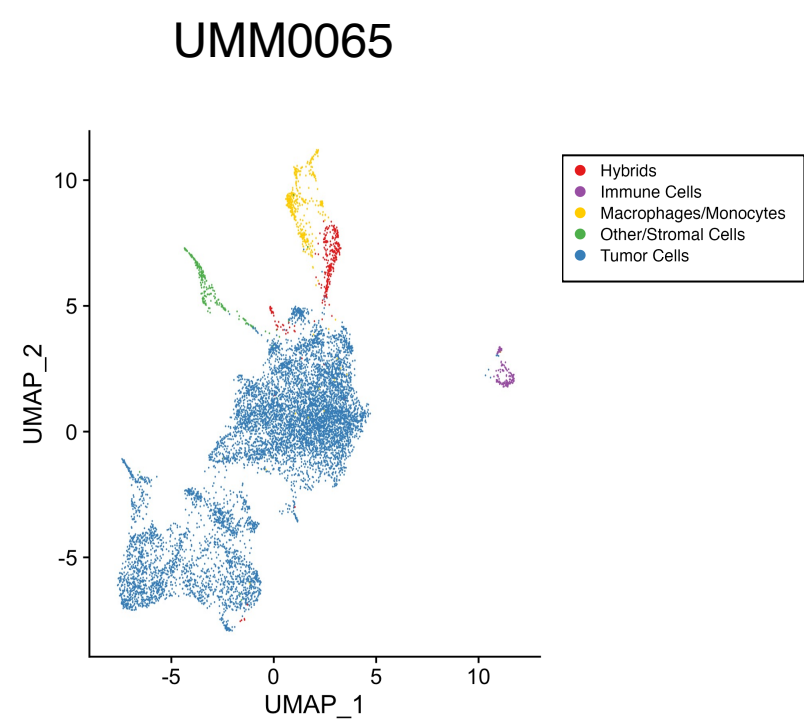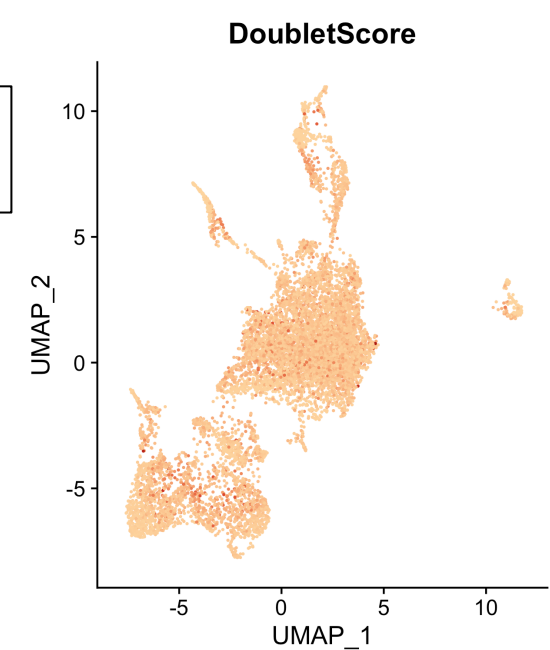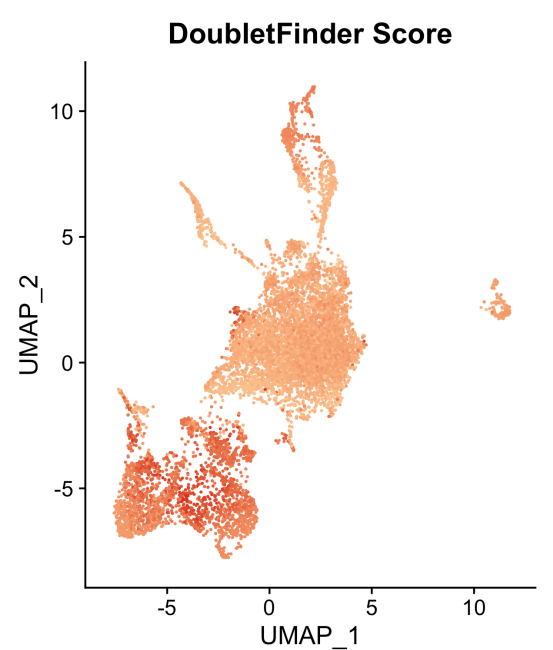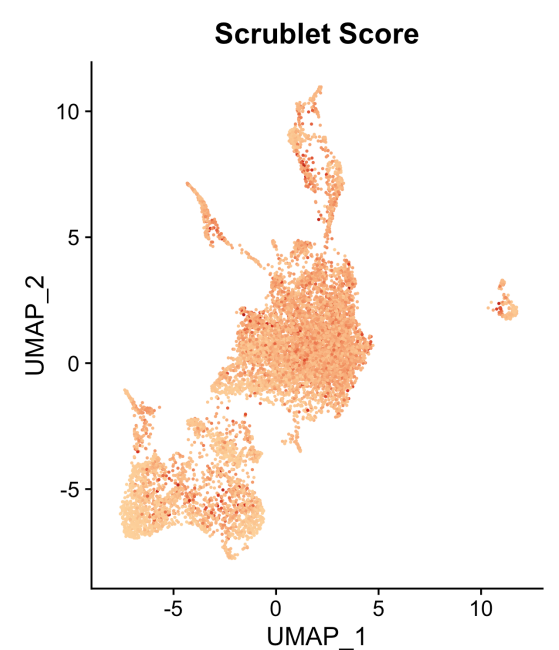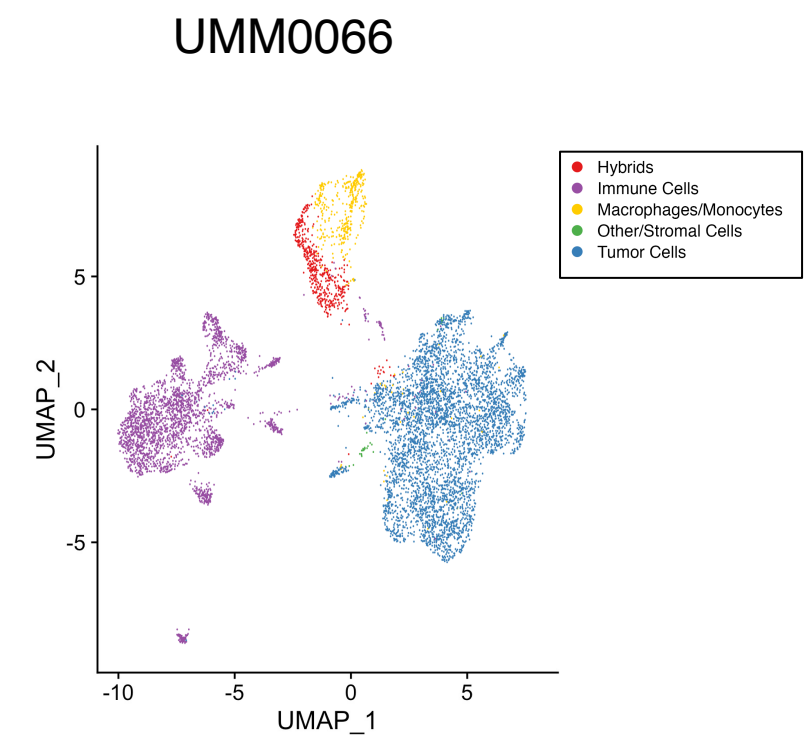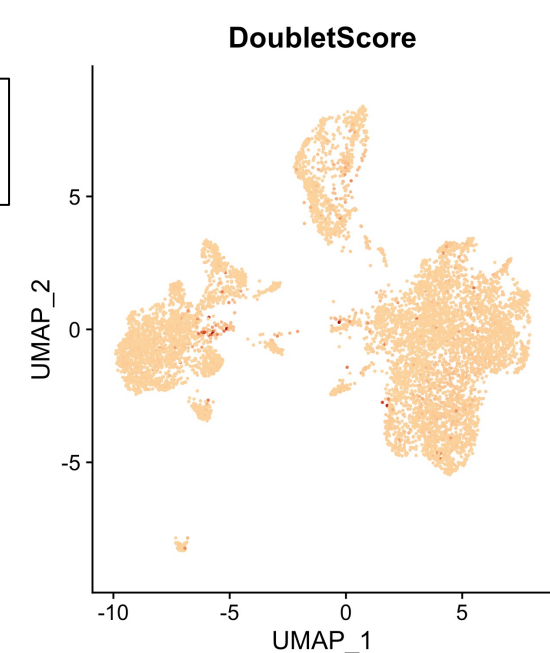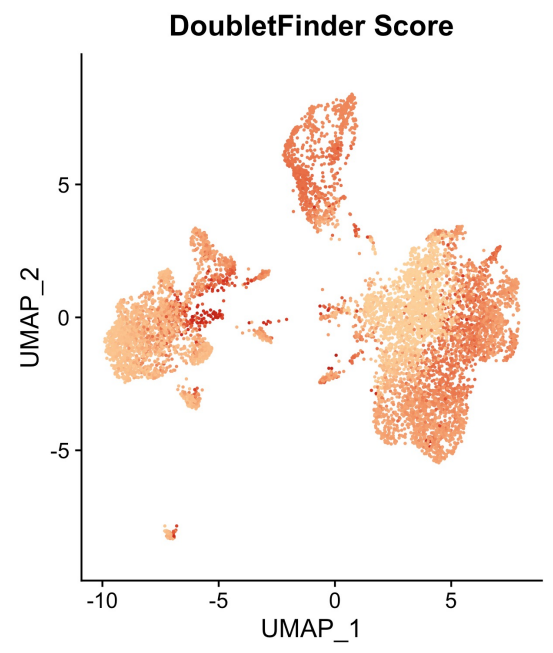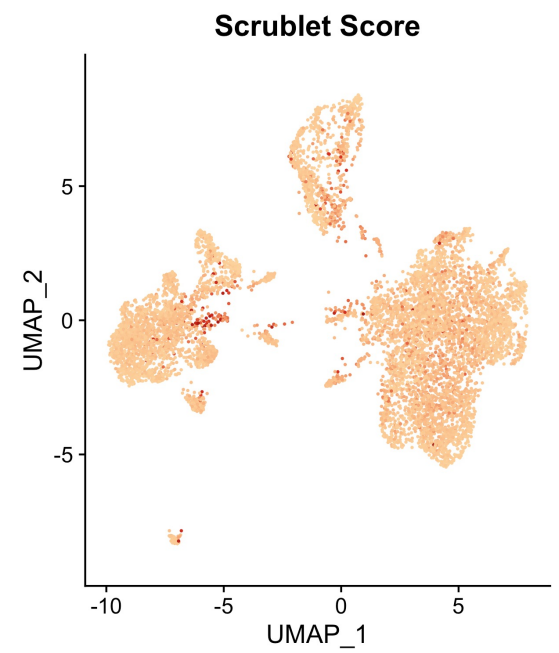

Supplement: Supplementary file 2 — Additional File 2: (UMM063-UMM066) ­– Hybrid cell identification from UM scRNA-seq dataset. A) Leiden-based clustering shown as a UMAP. B) Hierarchical-based clustering of scRNA-seq data annotated by cluster number and major cell type (tumor, macrophage/monocyte, immune, stromal/other) C) Leiden-based clustering shown as a UMAP with annotation by major cell type where identified hybrid cells are in red. D) Individual UMAPs for gene expression of melanocytic and macrophage/monocyte genes. E, F) Tumor score and macrophage/monocyte score violin plots for each cluster and colored according to major cell type, where hybrid cells have significantly higher tumor scores than other immune and stromal cell clusters (*= p-value 3.0E-07 UM0063, p-value ≤ 1.60E-14 UM0064, p-value≤ 5.8E-16 UM0065, p-value ≤ 4.60E-14 UM0066). Hybrid cells have significantly higher macrophage/monocyte scores than all other tumor cell clusters (*= p-value≤ 0.02 UM0063, p-value ≤ 2.0E-16 UM0064, p-value ≤ 0.0028 UM0065, p-value ≤1.60E-11 UM0066). [file 40364_2024_609_MOESM2_ESM.pdf]

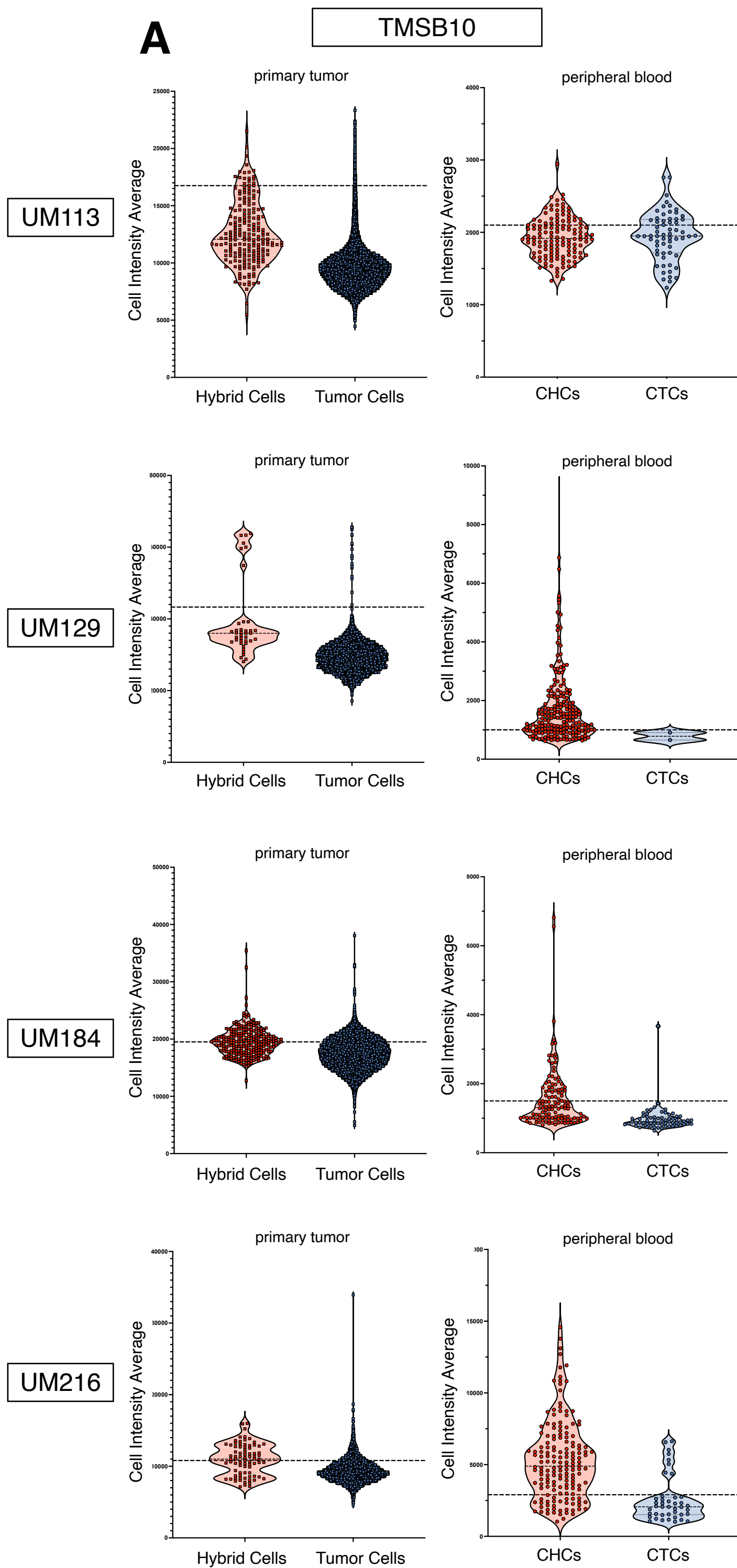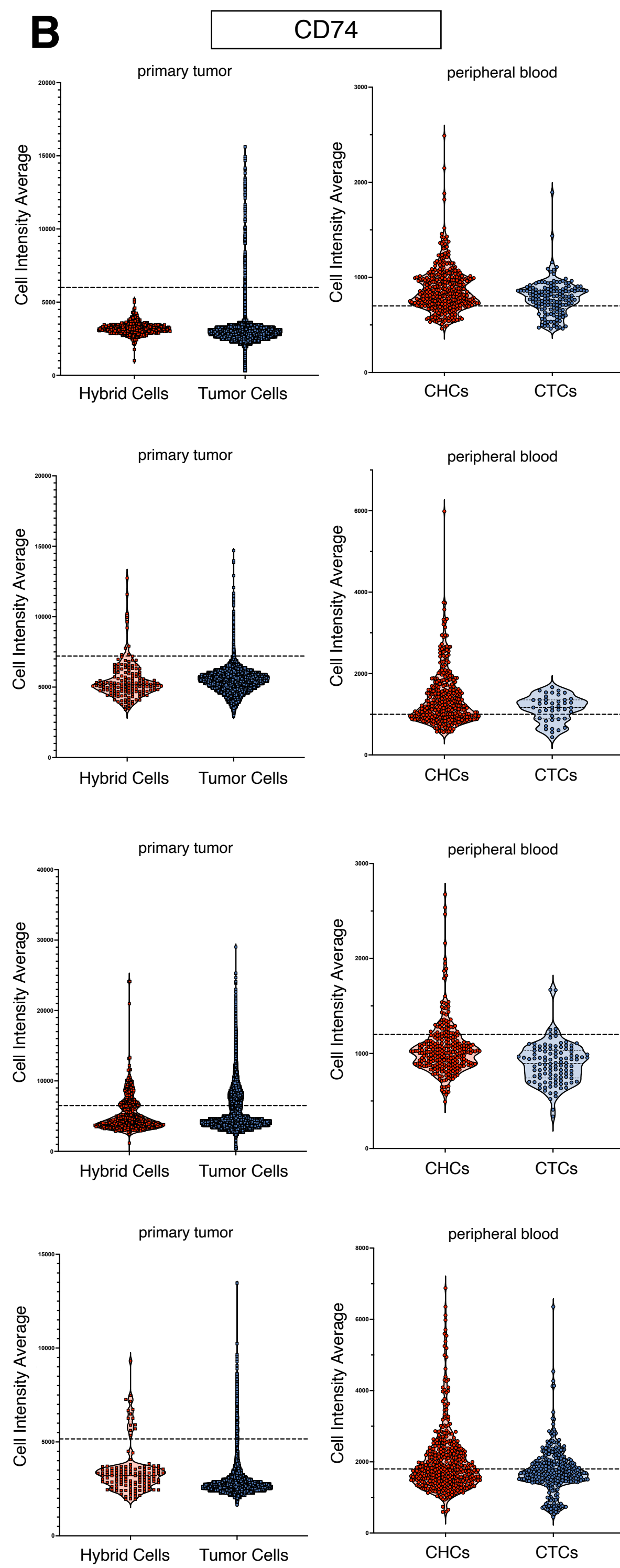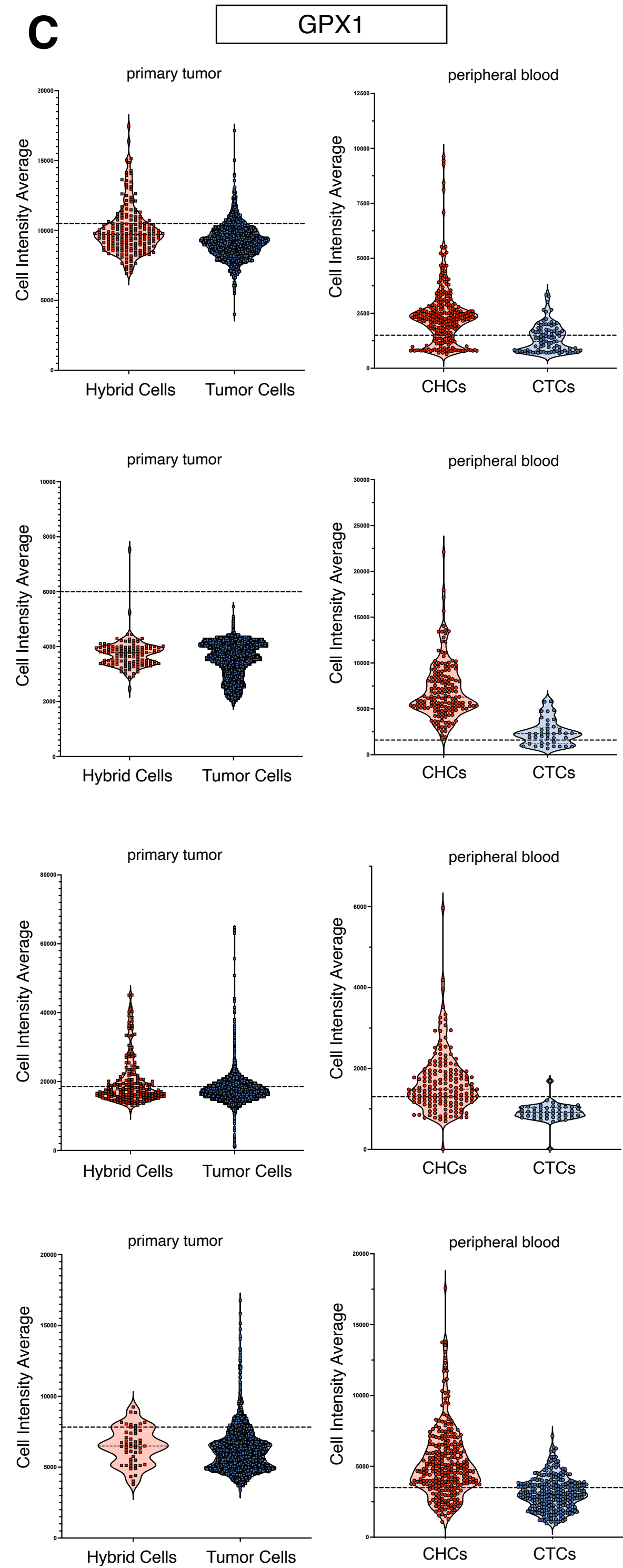

Supplement: Supplementary file 3 — Additional File 3: Hybrid cell distinction from predicted doublets for each patient. Doublet scores shown on each individual patient UMAP (A) for three doublet simulation methods; B) DoubletDensity (32), C) DoubletFinder (33), and D) Scrublet (34). [file 40364_2024_609_MOESM3_ESM.pdf]
